# Graphene Metamaterial Embedded within Bundt Optenna for Ultra-Broadband Infrared Enhanced Absorption

**DOI:** 10.3390/nano12132131

**Published:** 2022-06-21

**Authors:** Ehab Awad

**Affiliations:** Electrical Engineering Department, College of Engineering, King Saud University, Riyadh 11421, Saudi Arabia; esawad@ieee.org

**Keywords:** graphene, optical antenna, infrared plasmonics, nano-focusing, absorption enhancement

## Abstract

Graphene is well-known for its extraordinary physical properties such as broadband optical absorption, high electron mobility, and electrical conductivity. All of these make it an excellent candidate for several infrared applications such as photodetection, optical modulation, and optical sensing. However, a standalone monolayer graphene still suffers from a weak infrared absorption, which is ≅2.3%. In this work, a novel configuration of graphene metamaterial embedded inside Bundt optical-antenna (optenna) is demonstrated. It can leverage the graphene absorption up to 57.7% over an ultra-wide wavelength range from 1.26 to 1.68 µm (i.e., Bandwidth ≅ 420 nm). This range covers the entire optical communication bands of O, E, S, C, L, and U. The configuration mainly consists of a Bundt-shaped plasmonic antenna with a graphene metamaterial stack embedded within its nano-wide waveguide that has a 1.5 µm length. The gold average plasmonic loss is ≅25%. This configuration can enhance graphene ultra-broadband absorption through multiple mechanisms. It can nano-focus the infrared radiation down to a 50 nm spot on the graphene metamaterial, thus yielding an 11.5 gain in optical intensity (i.e., 10.6 dB). The metamaterial itself has seven concentric cylindrical graphene layers separated by silicon dioxide thin films, thus each layer contributes to the overall absorption. The focused infrared propagates tangential to the graphene metamaterial layers (i.e., grazing propagation), and thus maximizes the light–graphene interaction length. In addition, each graphene layer experiences a double-face exposure to the nano-focused propagating spot, which increases each layer’s absorption. This configuration is compact and polarization-insensitive. The estimated maximum absorption enhancement compared to the standalone monolayer graphene was 25.1 times (i.e., ≅4 dB). The estimated maximum absorption coefficient of the graphene stack was 5700 cm^−1^, which is considered as one of the record-high reported coefficients up to date.

## 1. Introduction

Graphene is an attractive material for photodetection, optical modulation, and optical sensing applications because of its broadband optical absorption, high electron mobility, and electrical conductivity [1]. The synthesis, fabrication, and processing techniques of graphene layers have become notably advanced in recent years [2], which is promising for practical use in many applications. However, it is well-known that the standalone monolayer graphene is still suffering from a weak optical absorption, which is estimated to be ≅2.3% over the visible and near-infrared bands [3]. One way to overcome this obstacle is to combine graphene with plasmonic noble metals in configurations that can enhance graphene broadband optical absorption, especially within the shortwave infrared range (1 to 2 µm). Of course, noble metal plasmonics has been an active research topic over the last decade [4]. Metal plasmonics can concentrate optical fields within a sub-wavelength nano spot in the metal vicinity with very high magnitudes, which in turn can increase the optical absorption of neighboring materials. An additional approach is to use a graphene multilayered metamaterial, and thus each layer can contribute to increasing the overall material optical absorption.

Different techniques have been reported thus far on manipulating the graphene optical absorption [5,6,7,8,9,10,11,12,13,14,15,16,17,18]. A polarization-sensitive electro-absorption modulator based on a waveguide-integrated monolayer graphene was demonstrated over the wavelength range of 1350 nm to 1600 nm with an electroabsorption modulation equal to 0.1 dB µm^−1^ [5]. A 90 nm thick graphene metamaterial absorber with a 12.5 cm^2^ area consisting of a large number of graphene layers and arranged in a grating device backed by a mirror showed an enhanced unpolarized light absorption of 85% over the solar-spectral bandwidth [6]. A CMOS-compatible and polarization-sensitive bilayer-graphene photodetector that was 24 µm-long showed an optical response over the optical communication bandwidth with a peak absorption of 44% at the wavelength of 1550 nm [7]. A plasmonic infrared antenna on top of a graphene photodetector was used to enhance the responsivity within the mid-infrared band with an enhanced optical absorption of 10% [8]. A graphene-based optoelectronic modulator of amplitude, phase, and polarization was theoretically demonstrated using graphene absorption [9]. A 5 µm-long plasmonic slot graphene photodetector with a high responsivity at 1310 nm was demonstrated [10], which achieved a graphene maximum absorption of ≅40% and an absorption coefficient of ≅2300 cm^−1^ at a slot gap of ≅15 nm. Critical coupling with guided Bragg-mirror resonance was used to achieve a narrow-band and polarization-sensitive complete absorption of ≅99% in the monolayer graphene [11]. A graphene photodetector integrated on a silicon microring resonator showed a high responsivity with ≅92% absorption in a 6 µm long graphene layer under critical coupling [12], which corresponded to an ≅4200 cm^−1^ absorption coefficient. A double layer graphene electro-absorption modulator on a silicon-on-insulator waveguide was demonstrated with an optical absorption of 0.13 dB µm^−1^ [13]. Complete optical absorption in a periodically patterned nanodisk graphene device was demonstrated using critical coupling [14]. Dart-type single-layer graphene was utilized to build a polarization-independent multi-mode surface plasmon resonance absorber with a metal reflector [15], which showed perfect absorption within the mid-infrared wavelength range. A nano-composite of mesoporous titanium dioxide doped by Ag-coated graphene showed an improved optical absorption within the visible light range [16]. Angle-selective perfect absorption showed a narrow band at 47.2% optical absorption in graphene [17]. A review of graphene-based perfect absorption structures was explored in [18].

In this work, a novel configuration of the graphene metamaterial embedded in a plasmonic waveguide [19] of Bundt optical antenna (optenna) is demonstrated. It can enhance the graphene ultra-broadband infrared absorption over a portion of the shortwave infrared range. The graphene metamaterial consists of a cylindrical stack that is embedded within a gold nanoplasmonic waveguide of a Bundt-shaped optenna [20]. The metamaterial stack consists of seven cylindrical and concentric graphene layers that are separated by 7 nm silicon dioxide thin films. The free-space infrared radiation is collected and coupled by a Bundt coaxial horn to excite surface plasmon polariton (SPP) traveling waves on the horn surface. The SPP is then nano-focused [21] by the horn tapered surface down to a 50 nm spot, where it can end-fire and excite a plasmonic annular waveguide with an embedded graphene stack. The graphene metamaterial absorption is maximized by four mechanisms. First, the plasmonic subwavelength nano-focusing of infrared radiation yields a high gain (10.6 dB) of infrared intensity. Second, using several graphene layers with each one contributes to increasing the overall absorption. Third, the tangential (i.e., grazing) propagation of the nano-spot along the graphene metamaterial layers maximizes the light–matter interaction length. Fourth, the alternating layers of graphene and silicon dioxide thin films allow for a double-face exposure of each graphene layer to a nano-focused infrared spot. This configuration shows several advantages. It is polarization-insensitive. It can leverage graphene metamaterial absorption up to 57.7%, and the absorption coefficient of it up to 5700 cm^−1^. The absorption enhancement compared to standalone monolayer graphene can reach up to 25.1 times (i.e., ≅14 dB). The enhanced absorption has an almost continuous response over all the optical communication bands: O (1260 to 1360 nm), E (1360 to 1460 nm), S (1460 to 1530 nm), C (1530 to 1565 nm), L (1565 to 1625 nm), U (1625 to 1675 nm), with an overall bandwidth of ≅420 nm. This device configuration is promising for free-space infrared optical communications with applications in photodetection, optical modulation, monitoring, and sensing.

## 2. Operation Principles

**Figure 1:**
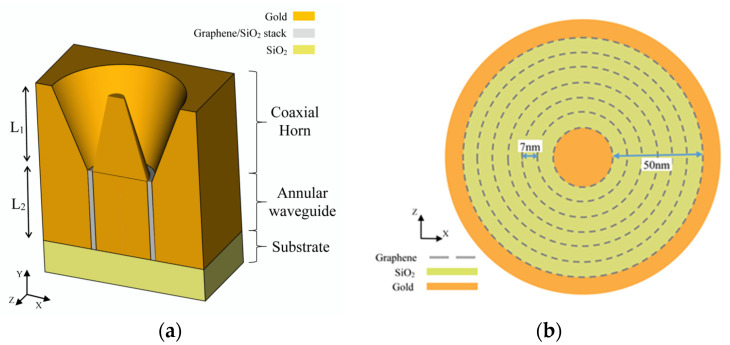
The graphene metamaterial embedded Bundt optenna device configuration. (**a**) A three-dimensional schematic diagram of the device with an XY cross section showing different stages. (**b**) A magnified XZ cross section in the waveguide with a cylindrical graphene metamaterial stack showing the alternating and concentric silicon dioxide thin films and graphene layers.

Figure 1a shows a cross section in the proposed configuration of graphene metamaterial embedded within the Bundt optenna. The plasmonic Bundt optenna is made of gold [20], and it consists of two stages that are built on a silicon dioxide substrate. The first stage is a tapered coaxial horn with a 2 µm length (L_1_). At the horn input, the inner and outer diameters are 0.05 and 1.4 µm, respectively, whereas, the inner and outer diameters at the horn output are 0.7 µm and 0.8 µm, respectively, resulting in an annular 50 nm-wide gap. The second stage is a straight waveguide that is 50 nm wide and 1.5 µm long. The graphene metamaterial annular stack is embedded to fill the nano waveguide width. The stack consists of seven concentric cylindrical graphene sheets separated by 7 nm silicon dioxide thin films. The first and last graphene layers are in contact with the waveguide gold inner and outer surfaces. Of course, more graphene layers would enhance the metamaterial absorption further, however, seven layers were enough to fill up the 50 nm-wide waveguide, given that the minimum standard width of the separating thin films is 7 nm. Figure 1b shows a magnified XZ cross section within the waveguide illustrating the cylindrical graphene metamaterial with alternating concentric silicon-dioxide thin films and graphene layers. This device is polarization-insensitive because of the circular symmetry around its central axis. The tapered coaxial horn collects input infrared radiation from free space and excites SPP on the gold surfaces, which are coupled together across the horn gap. The horn squeezes the plasmonic fields gradually down to a sub-wavelength 50 nm-wide spot at the input of the annular plasmonic waveguide. The reduction in the plasmonic field area results in an optical intensity gain. This focused guided infrared plasmonic field propagates along the longitudinal direction of the cylindrical graphene layers until it becomes fully absorbed by the end of the waveguide. The infrared spot propagates tangentially along and in between the graphene layers. Thus, each layer is double-face exposed to focused infrared radiation during propagation.

The graphene metamaterial embedded Bundt optenna device was designed and simulated using the three-dimensional finite-difference time-domain (FDTD) method [22]. The FDTD is well-known as an efficient method for modeling plasmonic devices [23]. A unit-cell, which is shown in Figure 1a, is placed in a two-dimensional structure of a periodic square array with a periodicity of 1.425 µm. The graphene chemical potential was set to 0.1 eV, which was found to be the best value to have an ultra-wide absorption bandwidth covering all the optical communication bands. The device’s periodic structure was simulated using a one unit-cell after applying periodic-Bloch boundary conditions on the cell sidewalls. Stretched perfectly-matched boundary conditions were set on the top and bottom of the unit cell with a max of 256 layers for stabilized simulation. The FDTD simulation time was set to 1000 femtoseconds. The FDTD was performed using a non-uniform mesh with a maximum mesh step of 1 nm. The mesh accuracy was set to 2 with the default conformal mesh refinement, and a minimum mesh step size of 0.25 nm. The simulation temperature was set to room temperature (300 K). Convergence tests were conducted during the simulations to ensure very small numerical errors. A broad-band plane-wave source with linear polarization (either TM or TE) was utilized. The source wavelength was set within one of the tested bands. The tested bands were the O (1260 to 1360 nm), E (1360 to 1460 nm), S (1460 to 1530 nm), C (1530 to 1565 nm), L (1565 to 1625 nm), and U (1625 to 1675 nm), which covers all of the optical communication bandwidths.

The gold and silicon dioxide material models were selected from the software complex refractive indices library of Palik [24] whereas the graphene model was obtained from the software conductivity (*σ*) library, which is built around the well-known Kubo graphene model. The graphene complex refractive indices (*n*) are obtained using the formula:

$$n=\sqrt{\varepsilon_{r}+j\frac{\sigma }{\varepsilon_{o}\omega \;\Delta }}$$

where ‘*ε_r_*’ is relative permittivity; ‘*ε_o_*’ is free-space permittivity; ‘*ω*’ is the infrared angular frequency; ‘Δ’ is the graphene layer thickness, which is assumed here to be 1 nm. The graphene chemical potential was selected to be 0.1 eV. A multi-coefficient model was used to fit the materials’ complex indices.

**Figure 2:**
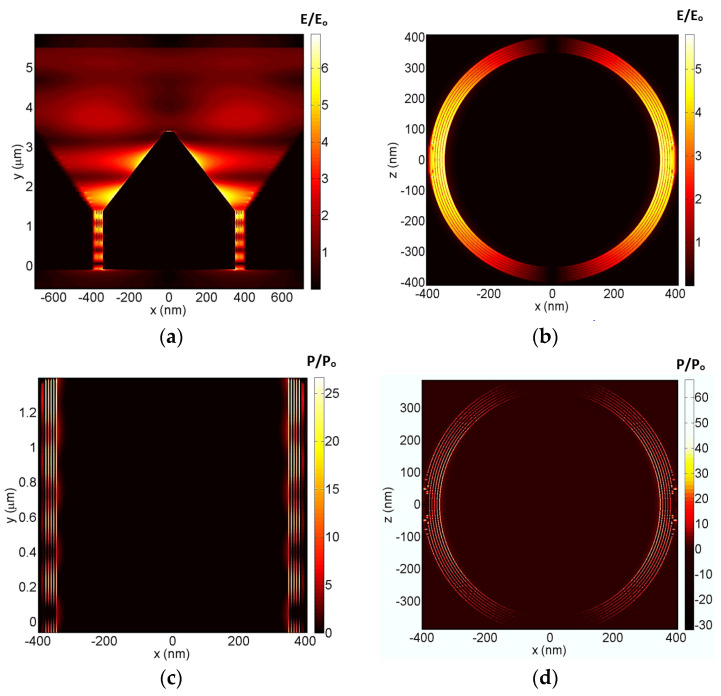
The three−dimensional finite−difference time−domain of normalized electric fields (E/E_o_) and absorption ratios (P/P_o_) at a wavelength of 1476 nm. (**a**) An XY vertical cross−section of the graphene metamaterial embedded Bundt optenna, (**b**,**d**) An XZ horizontal cross−section at the waveguide input, and (**c**) an XY vertical cross section of the waveguide.

Figure 2 shows the plasmonic fields and power absorption within different parts of the device in the case of TM-polarized incident radiation at a 1476 nm wavelength. This wavelength was selected as it yields a maximum metamaterial absorption, as will be discussed later in Section 3. The fields and power were normalized to the incident source field and power, respectively. Figure 2a illustrates the plasmonic field within an XY section of the whole device. It shows that infrared collection and focusing through the coaxial horn had high values in the vicinity of gold surfaces and the highest field values at the plasmonic waveguide entrance. Therefore, the inner and outer graphene layers were placed in contact with the waveguide gold surfaces to become exposed to these maximum plasmonic fields within the waveguide. The plasmonic field becomes almost negligible by the waveguide end due to complete absorption.

All of the Bundt optenna and the graphene metamaterial dimensions were optimized through numerical iterations to minimize the infrared back reflections and thus ensure the maximum input power to the device and in turn the maximum graphene absorption. The input coaxial horn dimensions were optimized to match the optical impedance of the free-space incident radiation. Additionally, the plasmonic waveguide dimensions were optimized to match the output optical impedance of the first stage coaxial horn. The length of the plasmonic waveguide (L_2_ = 1.5 µm) was maximized to achieve almost total absorption within the metamaterial. The waveguide acts as a Fabry–Perot due to multiple reflections between its input and output ports. Thus, the waveguide length was also optimized to have almost total destructive interference among the back-reflected waves at its input. In addition, the inner perimeter of the waveguide gap was selected to be equal to the maximum operating wavelength to sustain the mode propagating inside the waveguide. As seen in Figure 2a, the back-reflected field is almost negligible, which indicates a well optical impedance matching between the Bundt input and free space. This was achieved by optimization of the Bundt dimensions.

Figure 2b illustrates the normalized field magnitude of an XZ cross section at the waveguide input. It shows the fluctuating concentric field distribution around the graphene circular layers. Figure 2c illustrates the power absorption ratio within an XY cross section of the plasmonic waveguide. As seen, the absorption had the highest values inside the graphene layers, in addition to some absorption on the waveguide gold surfaces. Figure 2d illustrates the power absorption ratio of an XZ cross section at the plasmonic waveguide input. The artifacts at the X-boundaries are due to imperfections of the utilized absorption monitor in the close vicinity of the outer graphene layer. It is worth mentioning that the same values of the plasmonic fields and power absorption were obtained for the case of TE-polarization, indicating a polarization-insensitive operation of the device due to its circular symmetry.

**Figure 3:**
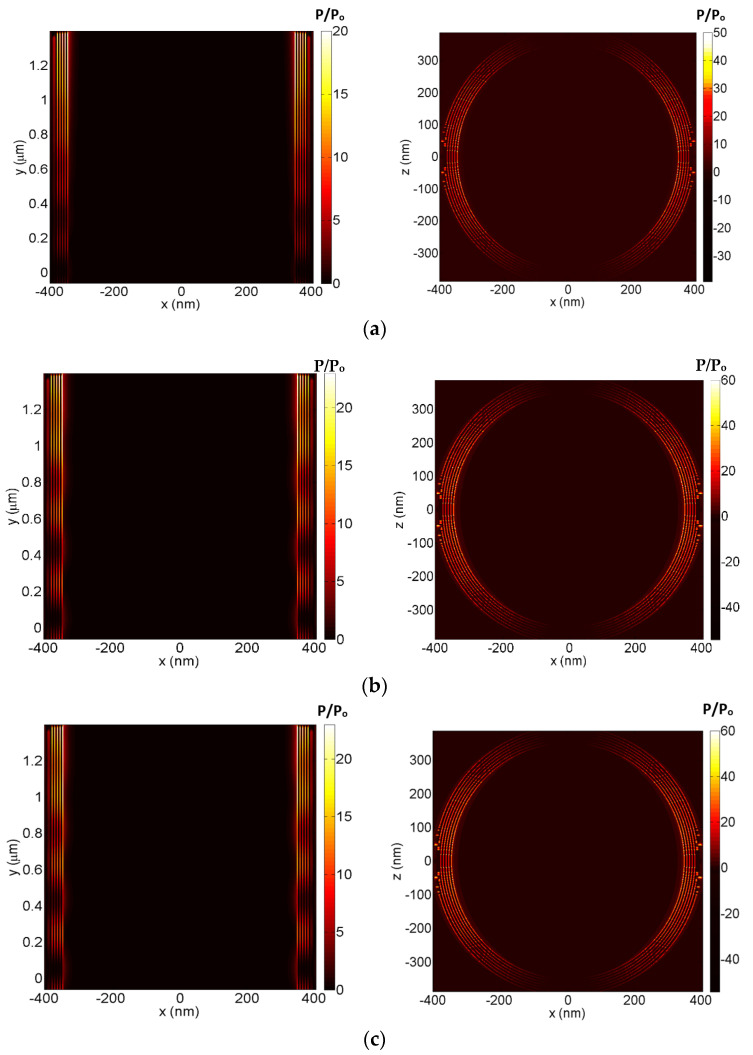
The three−dimensional finite−difference time−domain of the absorption ratios (P/P_o_) for the XY cross section and XZ cross section inside the plasmonic waveguide at some selected wavelengths: (**a**) 1300 nm, (**b**) 1600 nm, (**c**) 1439 nm.

Figure 3 shows the power absorption ratios at the same XY cross section and XZ cross section of the plasmonic waveguide, however, for the other wavelengths. Figure 3a–c corresponds to the wavelengths of 1300, 1600, and 1439 nm, respectively. As seen, the highest absorption was concentrated within the graphene layers with some plasmonic absorption on the waveguide gold surfaces. The wavelength of 1439 nm was selected as it corresponds to the minimum metamaterial absorption, as will be discussed later in Section 3.

## 3. Device Performance

The graphene metamaterial embedded Bundt optenna device configuration was also evaluated by the three-dimensional FDTD method. The evaluation was conducted over the ultra-broadband wavelength range of 1260 nm to 1680 nm, which includes the O, E, S, C, L, and U bands of optical communications. The graphene three-dimensional indices model was extracted to facilitate the calculation of total power absorption by integrating the power absorbed per unit volume (i.e., power density) over the volume of the 1 nm thick graphene layer. This was achieved by applying two volumetric monitors, one for the electric field and the other for the refractive index within the absorbing layer. The absorbed power density was then calculated by $\;\frac{\omega }{2}\;{\left|E\right|}^{2}\;Imag\left\{\varepsilon \right\}$ [22] where ‘|*E*|’ is the electric-field magnitude, and ‘*Imag*{*ε*}’ is the imaginary part of permittivity at the operating wavelength.

**Figure 4:**
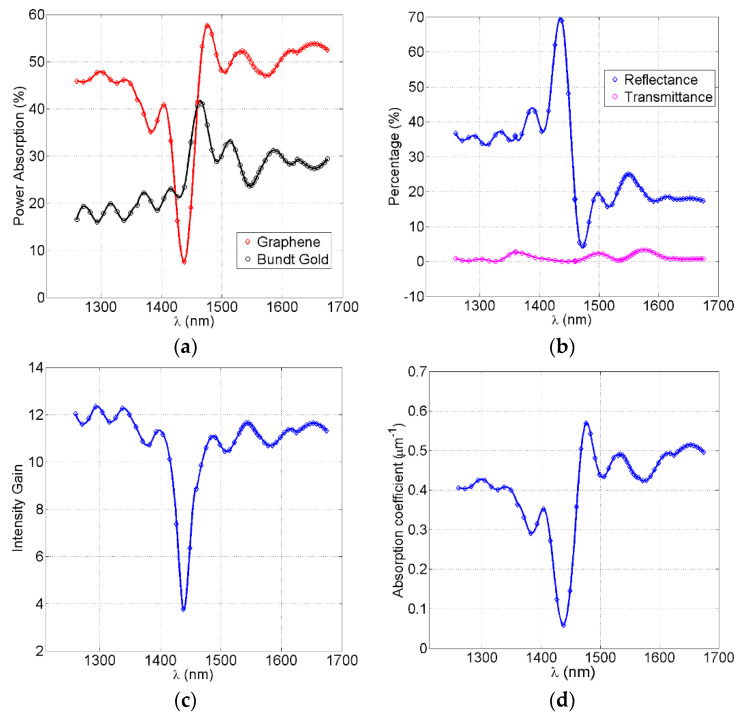
The performance evaluation of the graphene metamaterial embedded Bundt optenna device. (**a**) The percentage of incident power absorption in the graphene metamaterial and device gold surfaces. (**b**) The percentage of incident power reflected at the device input and transmitted at the device output. (**c**) The gain (i.e., enhancement) in infrared intensity at the output of the first−stage horn. (**d**) The absorption coefficient of the graphene metamaterial per micrometer length.

Figure 4a shows the percentage ratio of the absorbed power measured to incident power for each of the graphene metamaterials and the gold surface. The absorbed power in gold are the losses due to the surface plasmon polariton. It also includes plasmonic losses in the horn and waveguide sidewalls whereas the graphene stack absorption includes all of its seven layers. The graphene metamaterial absorption reached a maximum of 57.7% at a wavelength of 1476 nm, and a minimum of 7.9% at a wavelength of 1439 nm. This minimum was still more than the standard absorption of a standalone monolayer graphene. The gold absorption loss was always less than the graphene absorption by ≅20%, except in the graphene curve dip between 1425 nm and 1460 nm, where the gold losses overcame the graphene absorption. The average graphene metamaterial absorption was ≅45%, whereas the gold average absorption was ≅25%. The ripples on both curves were due to the Fabry–Perot effect resulting from multiple reflections between the plasmonic waveguide ends. The sharp dip in the graphene curve was due to Fabry–Perot-induced destructive interference at these particular wavelengths.

Figure 4b shows the percentage of back-reflected infrared at the device input as well as the percentage of transmitted infrared into the substrate at the waveguide output. Due to the absorption of the most infrared in the graphene metamaterial aside from some losses in gold, the infrared transmittance was almost zero. There was some back reflectance around 20% at longer wavelengths and 35% at shorter wavelengths because of some optical impedance mismatch between the free-space and input horn. The 70% spike in reflection at 1439 nm corresponded to the minimum dip of graphene absorption, where most of the incident infrared was reflected back due to the Fabry–Perot effect mentioned earlier. The minimum reflection of 4% at 1476 nm corresponded to the graphene maximum absorption.

Figure 4c illustrates the gain in infrared intensity due to coaxial feed horn squeezing of both the electric and magnetic plasmonic fields at the coaxial horn output and just before the plasmonic waveguide input. The horn area was downsized from 1.54 µm^2^ at the input to 0.11 µm^2^ at the output (i.e., plasmonic waveguide input). After subtracting the gold losses on the horn gold surface, the gain (i.e., enhancement) in the inferred intensity becomes ≅11.5 (i.e., 10.6 dB). Still, the dip in the curve (3.8%) corresponds to the maximum back reflection and thus yields a minimum graphene absorption. The intensity gain at the waveguide input for the 1476 nm wavelength was almost 11 dB, which yielded a maximum absorption after interaction with the metamaterial. 

Figure 4d shows the linear absorption coefficient (α) of the graphene metamaterial per micrometer length of the plasmonic waveguide. This can be obtained using the formula I_out_ = I_in_ e^−αL^_2_, where ‘I_in_’ is the input intensity to the waveguide, ‘I_out_’ is the output intensity of the waveguide, and ‘L_2_′ is the metamaterial length that equals the plasmonic waveguide length. The maximum absorption coefficient was 5700 cm^−1^, which corresponds to the maximum graphene absorption. The average absorption coefficient was ≅4500 cm^−1^, which was considered as one of the record-high reported values of the graphene absorption coefficient. Still, the dip in the curve corresponded to the minimum graphene absorption and maximum back reflection.

**Figure 5:**
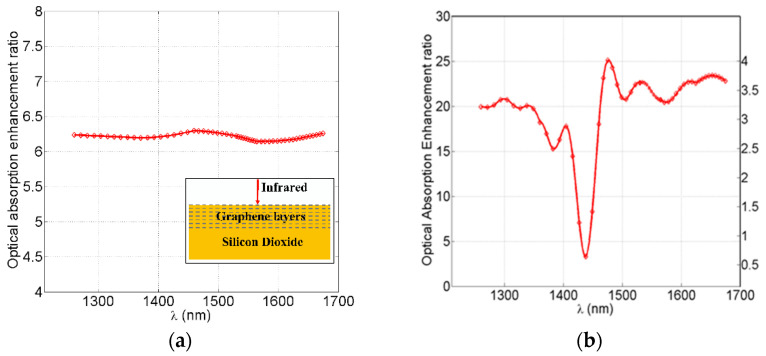
The optical absorption enhancement performance for different cases: (**a**) standalone graphene metamaterial (seven layers) with a perpendicular infrared incidence, as shown in the inset. The absorption was compared to standard standalone monolayer graphene, (**b**) graphene metamaterial Bundt optenna compared to the standalone metamaterial in case ‘a’ on the right axis, and compared to the standard standalone monolayer graphene on the left axis.

Figure 5 shows the optical absorption enhancement for different cases. Figure 5a shows the case when infrared radiation is incident perpendicular to a standalone graphene metamaterial layer without a gold Bundt optenna. This means that there are no gold SPP waves, no grazing incidence (tangential) propagation along the graphene layers, no infrared nano-focusing, and no double-face exposure of graphene layers to the infrared. The absorption enhancement, in this case, is the ratio between the optical absorbed power in the metamaterial and standard standalone monolayer graphene. The optical absorption enhancement here was due to the seven multilayers of graphene. The average enhancement value was 6.2 times (≅8 dB).

Figure 5b shows the optical absorption enhancement in the case of the graphene metamaterial with a Bundt optenna (presented in Figure 4a). In the figure’s right axis, it is compared to the previous case of the standalone metamaterial. The maximum enhancement was four times (≅6 dB) at a wavelength of 1477 nm. In the figure’s left axis, it was compared to the standard standalone monolayer graphene layer. The maximum enhancement here was 25.1 times (≅14 dB) at a wavelength of 1477 nm.

**Figure 6:**
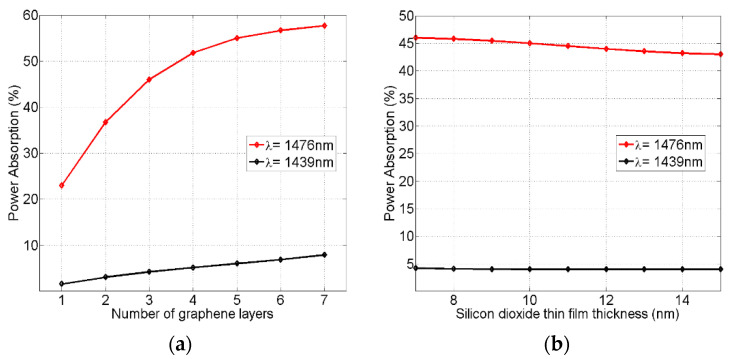
The variations in the metamaterial absorbed power ratio as a function of the number of concentric graphene layers (**a**), and the thickness of silicon dioxide (SiO_2_) thin films (**b**). The selected wavelengths of 1476 nm and 1439 nm corresponded to the maximum and minimum graphene absorption ratios in Figure 4a, respectively.

Figure 6 shows the variations in the metamaterial absorbed power performance as a function of the number of concentric graphene layers (Figure 6a) and the thickness of the silicon dioxide (SiO_2_) separating thin films (Figure 6b). The selected critical wavelengths were 1476 nm and 1439 nm, which corresponded to the maximum and minimum graphene power absorption ratios in Figure 4a, respectively. In Figure 6a, the metamaterial graphene layers varied from one up to seven layers while keeping the SiO_2_ thin film thickness fixed at 7 nm. As seen, the number of layers is very critical for the maximum power absorption measured at a 1476 nm wavelength. However, it had a smaller effect on the minimum power absorption at a 1439 nm wavelength. Thus, and as expected, the more graphene layers are added, the better the absorption ratio obtained.

In Figure 6b, the SiO_2_ thin film thickness was varied from a minimum standard value of 7 nm up to 15 nm while keeping the number of graphene layers fixed at three layers. The choice of three graphene layers here allowed for the metamaterial to accommodate the 50 nm gap width of the nano waveguide while the SiO_2_ thin film thickness increased up to 15 nm. As seen, the thin film thickness had a small effect on the maximum power absorption measured at the 1476 nm wavelength, whereas it had a negligible effect on the minimum power absorption at a 1439 nm wavelength. Therefore, these figures indicate that the best absorption performance of the metamaterial can be achieved by adding more graphene layers and reducing the thickness of the silicon dioxide thin films.

## 4. Conclusions

A novel configuration to enhance the ultra-broadband infrared absorption coefficient in a graphene metamaterial using a Bundt optical antenna was numerically demonstrated. The graphene metamaterial stack consisted of seven layers of separated cylindrical graphene layers with a length of 1.5 µm. The antenna can nano-focus infrared radiation and propagate it tangential to each graphene layer, thus allowing for a double-face exposure. This polarization-insensitive configuration gives a 10.6 dB intensity gain and enhanced the graphene metamaterial absorption of up to 57.7%. The estimated maximum absorption coefficient of the graphene metamaterial was 5700 cm^−1^, with an average of ≅4500 cm^−1^, which is considered among one the record-high reported coefficients to date. The device has a compact size and ultra-broadband response (≅420 nm), covering the entire optical communication bands (O, E, S, C, L, and U). The configuration of the graphene-embedded Bundt optenna is promising for free-space infrared optical communications with applications in photodetection, optical modulation, monitoring, and sensing.

Although this work focused mainly on the numerical simulations of graphene metamaterial embedded within a Bundt optenna, it is worth mentioning a hint on one suggested method for fabrication. A unit cell can be fabricated from the inside out. The chemical vapor deposition (CVD) method can be used to fabricate a cylindrical gold rod with a diameter of 0.7 µm and a total length of 3.5 µm (L_1_ + L_2_), which constitutes the Bundt central post. The CVD can be used to deposit alternating layers of graphene and silicon dioxide of metamaterial on the rod bottom surface with a length of 1.5 µm. After that, CVD can be used to deposit more gold onto the metamaterial together with the entire rod sidewalls until reaching the full width of a unit cell. Then, the concentric conical horn can be formed inside the gold using focused ion beam milling while controlling the milling area by changing the beam current and etching time.

The fabricated device samples could be tested experimentally with different methods. One suggested method is micro-FTIR (Fourier transform infrared) spectroscopy. The fabricated Bundt optenna samples without embedded graphene metamaterial can be tested with micro-FTIR to measure the optical absorption spectra across different wavelength ranges. Next, other fabricated Bundt optenna samples with the embedded graphene metamaterial can be tested to measure the difference in the optical absorption spectra over the same wavelength bands, and thus estimate the graphene metamaterial absorption by itself. Another method is to use a scattering-type scanning near-field optical microscope (SNOM) that is combined with a pseudo-heterodyne detection module, which can image the scattered near-fields of the micro- and nanostructures at a very small spatial resolution as small as 10 nm. The SNOM can map the scattered electric fields from the fabricated Bundt optenna samples with/without embedded graphene metamaterial, similar to extracting the optical power absorption by the Bundt gold material and then the graphene metamaterial.

## Data Availability

The author confirms that all of the data supporting this study’s findings are available within the article.
